# Postoperative Long-Term Outcomes and Independent Risk Factors of Non-Small-Cell Lung Cancer Patients With Propofol versus Sevoflurane Anesthesia: A Retrospective Cohort Study

**DOI:** 10.3389/fphar.2022.945868

**Published:** 2022-07-22

**Authors:** Zhenglian Gao, Jian Xu, Mark Coburn, Daqing Ma, Kun Wang

**Affiliations:** ^1^ Department of Anesthesiology, Harbin Medical University Cancer Hospital, Harbin, China; ^2^ Department of Anesthesiology, Panzhihua Central Hospital, Panzhihua, China; ^3^ Department of Anesthesiology, Cangzhou Central Hospital, Cangzhou, China; ^4^ Department of Anesthesiology and Intensive Care Medicine, University Hospital Bonn, Bonn, Germany; ^5^ Anaesthetics, Pain Medicine and Intensive Care, Department of Surgery and Cancer, Faculty of Medicine, Imperial College London, Chelsea and Westminster Hospital, London, United Kingdom; ^6^ Department of Anesthesiology, The First Affiliated Hospital of Harbin Medical University, Harbin, China

**Keywords:** propofol, sevoflurane, survival, risk factors, non-small-cell lung cancer

## Abstract

**Background:** Existing studies have shown that the relationship between anesthetic agents and non-small-cell lung cancer (NSCLC) prognosis remains controversial. Therefore, this retrospective cohort study was designed to investigate the effects of propofol or sevoflurane anesthesia on the long-term oncologic outcomes of NSCLC patients.

**Methods:** We identified 1,778 eligible patients (propofol-based total intravenous anesthesia (TIVA) group, *n* = 686; sevoflurane-based inhalation anesthesia (INHA) group, *n* = 1,092) out of 2,388 patients undergoing elective NSCLC surgery from June 2013 to June 2016 in the Harbin Medical University Cancer Hospital. The primary endpoints were five-year overall survival and recurrence-free survival. The secondary endpoints were independent risk factors of cancer recurrence or all-cause mortality. The data were analyzed with propensity score matching, Kaplan–Meier survival, and Cox multivariate analyses as appropriate.

**Results:** After propensity score matching, there were 672 patients in each group. The median follow-up period was 69 months (interquartile range: 68–70 months) for all patients. Five-year overall survival was 75.7% (95% confidence interval (CI) 72.4–79.1) in the TIVA group and 71.8% (68.4–75.4) in the INHA group (*p* = 0.160) (hazard ratio (HR), 0.86; 95% CI, 0.70–1.06; *p* = 0.158), and five-year recurrence-free survival was 68.5% (65.0–72.2) and 62.7% (59.1–66.5 (*p* = 0.108) (HR, 0.90; 95% CI, 0.75–1.08; *p* = 0.253), respectively. Subgroup analyses showed there were no significant difference in the overall survival or recurrence-free survival between the two groups in each TNM stage of NSCLC. The independent risk factors included age ≥60 years, male, blood transfusion, segmental/wedge resection and pneumonectomy, thoracotomy, postoperative complications, lung adenocarcinoma, TNM stages, high CEA and CYFRA211 levels, and postoperative radiotherapy.

**Conclusions:** Our data indicated no difference between the propofol-based TIVA and sevoflurane-based INHA in terms of five-year overall survival and recurrence-free survival after NSCLC surgery.

## Introduction

Lung cancer has the highest incidence and mortality among all malignant cancers worldwide (Sung et al., 2021) and its most common pathological type is non-small cell lung cancer (NSCLC), accounting for 85% of the total diagnosed cases (Oser et al., 2015). Although recent advances in therapeutic strategies, including surgery, radio- and targeted immune-therapy (Horowitz et al., 2015), the five-year survival of the operable NSCLC including all stages is still not optimal (Lu et al., 2019). This is likely due to the malignant nature of disease but risk factors including certain anesthetics/techniques use during the perioperative period that contributed cancer reoccurrence and patients’ death is emerging. Indeed, inhaled anesthetic isoflurane has been reported to increase hypoxia inducible factor (HIF), promoting angiogenesis through vascular endothelial growth factor (VEGF) signaling (Benzonana et al., 2013), likely accelerating cancer progression. The markers and mediators of angiogenesis, migration, invasion, proliferation, and even chemoresistance have been shown to be significantly enhanced by isoflurane through the potentiation of the tumorigenic PI3K/Akt/mTOR cell signaling pathway and upregulation of HIFs with these effects sustained for as long as 24 h after 2 h anesthetic exposure (Benzonana et al., 2013; Huang et al., 2014; Luo et al., 2015). In contrast, propofol has been shown to antagonize these same signaling pathways, as evidenced by a reduction in cellular levels of phosphorylated-Akt and HIF-1α protein synthesis. Furthermore, propofol also suppressed isoflurane’s aforementioned promalignant effects *in vitro* (Huang et al., 2014). Interestingly, propofol also encourages an anticancer environment by exerting little influence on NK cell and decreasing the synthesis of prostaglandin E_2_, a hormone that suppresses T-cell immunity (Inada et al., 2011). HIFs are ubiquitously expressed, evolutionarily conserved transcription factors that govern the cellular response to oxygen by activating transcriptional programs that promote adaptation and survival in conditions of hypoxia. It has been suggested that the HIF system plays a central role in cancer development (Unwith et al., 2015). In fact, tumors with high levels of HIF-1α and HIF-2α are significantly more malignant, metastatic, and resistant to radio- and chemo-therapy and are associated with a poorer prognosis (Semenza, 2003).

Furthermore, propofol at clinical concentrations regulated the biological behaviors of cancer cells, such as the proliferation, adhesion, invasion, metastasis, and tumor angiogenesis by mediating p38 MAPK, Wnt/β-catenin, and mTOR signaling pathways, and by upregulating or downregulating the expression of miR-372, lncRNA ANRIL, and circ-ERBB_2_ (Semenza, 2003; Zheng et al., 2020; Gao et al., 2021). In contrast, volatile anesthetics, such as isoflurane and sevoflurane promote the proliferation and migration of cancer cells *in vitro* (Benzonana et al., 2013), and increase the tumor load *in vivo* (Zhu et al., 2016). Sevoflurane directly suppresses cytokine release and the cytotoxicity of natural killer cells in experimental models, and potentially reduces the ability to destroy cancerous cells, which might increase the incidence of postoperative recurrence or metastasis (Jeon et al., 2020). In potentially keeping with this, a very recent clinical study has shown an association between volatile inhalational anesthesia and reduced long-term survival in cancer patients undergoing elective surgery (Wigmore et al., 2016). It must be stressed that the evidence available remains inadequate to substantiate any recommendations to alter the current clinical practice and more clinical study including trials are urgently needed (Perry et al., 2019).

Thus, we carried out a retrospective cohort study to assess whether the choice of anesthetics, propofol versus sevoflurane, influences the long-term survival after NSCLC surgery. Other risk factors related to postoperative recurrence or death were also analyzed in this study.

## Methods

### Study Design and Population

This retrospective cohort study is in compliance with the guidelines of the Declaration of Helsinki, and was approved by the Ethics Committee of Harbin Medical University Cancer Hospital. The informed consent was waived due to the study nature of the retrospective electronic medical record review and the data analyzed anonymously.

The inclusion criteria were patients who underwent an elective NSCLC surgery with age ≥18 years old, no distant metastasis assessed preoperatively, and the American Society of Anesthesiologists (ASA) physical status of I to III. The exclusion criteria included: pathological stage M1 or N3, unknown pathological type of lung cancer, unknown pathological stage, other malignancy, benign tumor, incomplete medical records (including follow-up failure), other inhalational but not sevoflurane anesthetics used, multiple operations, death within one month after surgery and incomplete resection.

The type of anesthesia used for including NSCLC operation was with the preference of anesthesiologists in Harbin Medical University Cancer Hospital as routine clinical practice. According to the type of anesthesia, patients were divided into propofol-based total intravenous anesthesia (TIVA) group or sevoflurane-based inhalational anesthesia (INHA) group. In this study, the TIVA group was set as the case group and the INHA group as the control group. Patients in both the groups were induced with 0.3 μg/kg sufentanil and 1–2.5 mg/kg propofol. In addition to sedatives and muscle relaxants, when necessary, the anesthesia maintenance was propofol 3 mg/kg/hr or sevoflurane 1–3% in combination with remifentanil 0.1–0.2 μg/kg/min. The INHA group was only given propofol during the induction period. The TIVA group received target-controlled infusion of propofol and remifentanil for anesthesia maintenance. All patients were treated with patient-controlled intravenous analgesia with a total 300 ml mixture of sufentanil (0.5 ug/mL) and flurbiprofen axetil (1 ug/mL) for postoperative pain control, and did not use additional regional anesthesia.

### Measurements

The data were harvested from the electronic medical records in patients who had lung cancer surgery in the Harbin Medical University Cancer Hospital between June 2013 and June 2016. Those were including age, sex, body mass index (BMI), smoking status, comorbidities at admission (hypertension, diabetes, cardiovascular disease, or cerebrovascular diseases), ASA physical status, anesthetics, intraoperative use of various drugs [dexmedetomidine, vasoactive drugs, opioids, or nonsteroidal anti-inflammatory drugs (NSAIDs)], perioperative blood transfusion, the type of operation (segmental/wedge resection, lobectomy, or pneumonectomy), video-assisted thoracic surgery (VATS), operation time, and complications after surgery. Tumor histological types (adenocarcinoma, squamous cell carcinoma, or other), tumor size, tumor-node-metastasis (TNM) cancer stages, tumor markers [squamous cell carcinoma antigen (SCC), carcinoembryonic antigen (CEA), and Cytokeratin-19 fragment 21-1 (CYFRA211)], chemotherapy or radiotherapy, cancer recurrence, and cancer related death were also recorded. The postoperative complications included anastomotic leakage, wound infection, pneumonia, malignant arrhythmia, acute myocardial infarction, heart failure, pulmonary embolism, cerebral infarction or hemorrhage, and acute renal failure. As more than 76% of patients were aged between 50 and 69 years old, patients were divided into four age groups (≤49, 50–59, 60–69, and ≥70 years old). According to the World Health Organization classifications, the BMI was divided into four categories (＜ 18.5 kg/m^2^, 18.5–24.9 kg/m^2^, 25.0–29.9 kg/m^2^, and ≥30.0 kg/m^2^). Because the number of patients with ASA grade I (*n* = 6) and III (*n* = 12) accounted for only 1% of the total, all patients were regarded as ASA grade II. All patients in this study received opioids for pain control and, thus, opioids were not included in the analysis as a variable.

### Study End-Points

The primary endpoints were five-year overall survival and recurrence-free survival after NSCLC surgery. The overall survival was defined as the period from the date of operation to the date of death or the last follow-up. Recurrence-free survival was defined as the period from the date of surgery to the date of recurrence, death, or the last follow-up. The dates of death and recurrence were obtained by the patients themselves or their relatives in the follow-up center of the Harbin Medical University Cancer Hospital. The deadline for the follow-up was 30 April 2021. Therefore, the duration and median of follow-up was between five and eight years. The secondary endpoints were independent risk factors for cancer recurrence or all-cause mortality.

### Statistical Analysis

The sample size of this study was the data from all patients undergoing NSCLC surgery in the Harbin Medical University Cancer Hospital from June 2013 to June 2016.

The data were described as the number with percentage for categorical variables and the mean with standard deviation or median with interquartile range for continuous variables, where appropriate. Shapiro–Wilk test was used to evaluate the normality of the distribution of continuous variables. The differences between the groups of continuous variables were compared with the independent sample t test or Wilcoxon rank sum test and the categorical variables were compared with the chi-square test.

The potential confounding effects of variables and the difference of baseline characteristics between the two groups were reduced by propensity score matching. Logistic regression analysis was used to estimate the propensity score of patients in the TIVA group. Two groups of patients were matched at a ratio of 1:1 by the nearest neighbor method with a caliper of 0.2 and an order of the largest propensity value (m.order = largest). The matched variables included age, sex, BMI, smoking status, hypertension, diabetes, cardiovascular disease, cerebrovascular disease, dexmetomidine, vasoactive drugs, NSAIDs, perioperative blood transfusion, type of operation, VATS, operation time, complications after surgery, tumor histologic types, TNM stages, SCC, CEA, CYFRA211, chemotherapy, and radiotherapy. The standardized mean difference (SMD) was used to evaluate the balance on baseline characteristics between the two groups when it was ＜ 0.10, indicating a good balance.

In the propensity-matched cohort, Kaplan–Meier survival curve was used to evaluate the overall survival rate and recurrence-free survival rate, and the log-rank test was used to compare the two groups. The median follow-up time was calculated by Reverse Kaplan–Meier method. The Cox proportional hazard regression models were utilized to estimate the relationship between the anesthesia type and overall survival or recurrence-free survival, and to determine the independent risk factors for NSCLC recurrence or all-cause mortality. The results were expressed as hazard ratio (HR) and 95% confidence interval (CI). The variables with *p* < 0.05 in the results of univariate Cox regression analysis, which included all variables and other potential risk factors, were adjusted by the multivariate Cox regression analysis. Complementary log plots and Schoenfeld residuals test were used to evaluate the proportional hazard assumptions. In addition, subgroup analyses were performed for the TNM stages of NSCLC to evaluate the association between anesthesia type and cancer prognosis.

All analyses were performed using R software version 4.1.2 (R Foundation for Statistical Computing, Austria). A two-tailed *p* < 0.05 was considered to be statistically significant.

## Results

**FIGURE 1:**
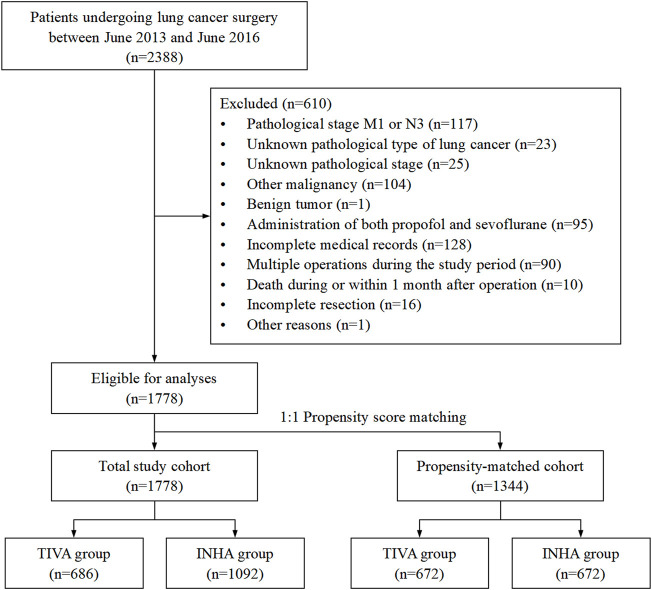
Flow diagram of the study population.

Of 2,388 patients undergoing NSCLC surgery during the study period at the Harbin Medical University Cancer Hospital, 1,778 patients (TIVA group, *n* = 686 and INHA group, *n* = 1092) were finally included in the analyses (Figure 1). The median follow-up period was 69 months (interquartile range: 68–70 months).

**FIGURE 2:**
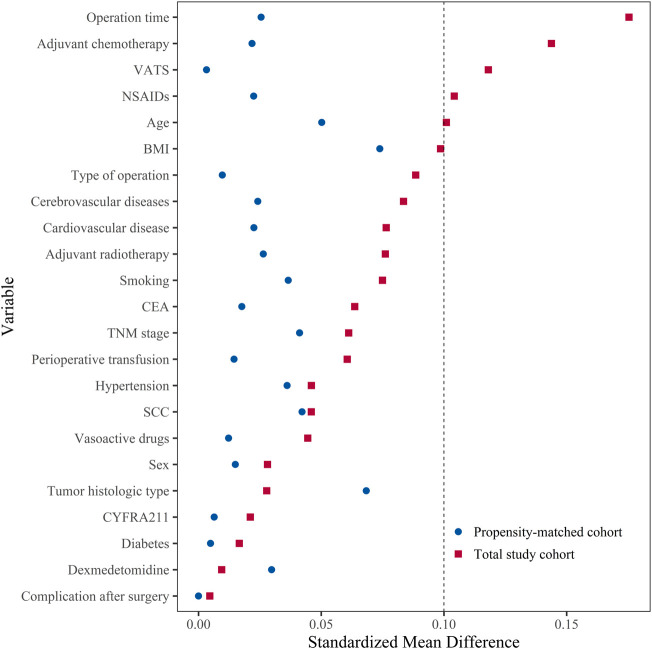
The distribution of standardized mean difference for variables included before and after matching. Standardized mean difference values <0.10 presented that the variable was well-matched and there was no difference between two groups. BMI, body mass index; CEA, carcinoembryonic antigen; CYFRA211, cytokeratin-19 fragment 21-1; NSAIDs, non-steroidal anti-inflammatory drugs; SCC, squamous cell carcinoma antigen; TNM, tumor-node-metastasis; VATS: video-assisted thoracic surgery.

The baseline characteristics of patients in the two groups for the total study cohort and the propensity-matched cohort (Table 1). In the total study cohort, there were differences between two groups in age, using NSAIDs drugs used, VATS, operation time, and adjuvant chemotherapy (SMD ≥0.10). After propensity score matching, there were 672 patients in each group. The SMD suggested that all variables were well-matched between the two groups (SMD <0.10) (Figure 2).

**TABLE 1:** Patient characteristics before and after matching.

	Before matching			After matching^a^		
	INHA (*n* = 1092)	TIVA (*n* = 686)	*SMD* ^b^	INHA (*n* = 672)	TIVA (*n* = 672)	*SMD*
Age (years)			0.101			0.050
≤49	167 (15.3)	97 (14.1)		92 (13.7)	94 (14.0)	
50–59	438 (40.1)	257 (37.5)		252 (37.5)	255 (37.9)	
60–69	396 (36.3)	282 (41.1)		287 (42.7)	275 (40.9)	
≥70	91 (8.3)	50 (7.3)		41 (6.1)	48 (7.1)	
Sex (male)	604 (55.3)	389 (56.7)	0.028	375 (55.8)	380 (56.5)	0.015
BMI^c^ (kg/m^2^)			0.099			0.074
<18.5	43 (3.9)	37 (5.4)		32 (4.8)	31 (4.6)	
18.5–24.9	715 (65.5)	424 (61.8)		432 (64.3)	418 (62.2)	
25.0–29.9	304 (27.8)	209 (30.5)		198 (29.5)	207 (30.8)	
≥30.0	30 (2.7)	16 (2.3)		10 (1.5)	16 (2.4)	
Smoking	398 (36.4)	275 (40.1)	0.075	259 (38.5)	271 (40.3)	0.037
Comorbidities at admission						
Hypertension	250 (22.9)	144 (21.0)	0.046	151 (22.5)	141 (21.0)	0.036
Diabetes	106 (9.7)	70 (10.2)	0.017	69 (10.3)	70 (10.4)	0.005
Cardiovascular disease	109 (10.0)	85 (12.4)	0.076	86 (12.8)	81 (12.1)	0.023
Cerebrovascular diseases	91 (8.3)	74 (10.8)	0.084	74 (11.0)	69 (10.3)	0.024
Dexmedetomidine	552 (50.5)	350 (51.0)	0.009	330 (49.1)	340 (50.6)	0.030
Vasoactive drugs	444 (40.7)	264 (38.5)	0.044	261 (38.8)	257 (38.2)	0.012
NSAIDs^c^	1080 (98.9)	669 (97.5)	0.104	661 (98.4)	659 (98.1)	0.022
Perioperative transfusion	101 (9.2)	76 (11.1)	0.061	70 (10.4)	73 (10.9)	0.014
Type of operation			0.088			0.010
Segmental/wedge resection	14 (1.3)	17 (2.5)		13 (1.9)	13 (1.9)	
Lobectomy	963 (88.2)	596 (86.9)		589 (87.6)	587 (87.4)	
Pneumonectomy	115 (10.5)	73 (10.6)		70 (10.4)	72 (10.7)	
VATS^c^	275 (25.2)	209 (30.5)	0.118	202 (30.1)	201 (29.9)	0.003
Operation time (hour)	2.51 ± 0.86	2.67 ± 0.94	0.175	2.62 ± 0.93	2.64 ± 0.88	0.025
Complication after surgery	23 (2.1)	14 (2.0)	0.005	13 (1.9)	13 (1.9)	<0.001
Tumor histologic type			0.028			0.068
Adenocarcinoma	728 (66.7)	457 (66.6)		460 (68.5)	450 (67.0)	
Squamous cell carcinoma	313 (28.7)	193 (28.1)		171 (25.4)	188 (28.0)	
Other	51 (4.7)	36 (5.2)		41 (6.1)	34 (5.1)	
TNM^c^ stage			0.061			0.041
Ⅰ	568 (52.0)	377 (55.0)		371 (55.2)	369 (54.9)	
Ⅱ	247 (22.6)	142 (20.7)		129 (19.2)	139 (20.7)	
Ⅲ	277 (25.4)	167 (24.3)		172 (25.6)	164 (24.4)	
SCC^c^ (1–1.5 ng/ml)	151 (13.8)	106 (15.5)	0.046	93 (13.8)	103 (15.3)	0.042
CEA^c^ (0–5 ng/ml)	283 (25.9)	159 (23.2)	0.064	153 (22.8)	158 (23.5)	0.018
CYFRA211^c^ (0–3.3 ng/ml)	361 (33.1)	220 (32.1)	0.021	213 (31.7)	215 (32.0)	0.006
Adjuvant radiotherapy	115 (10.5)	57 (8.3)	0.076	61 (9.1)	56 (8.3)	0.026
Adjuvant chemotherapy	450 (41.2)	235 (34.3)	0.144	241 (35.9)	234 (34.8)	0.022

a Logistic regression analysis was used to estimate the propensity score of patients in TIVA group including all variables. The two groups of patients were matched at a ratio of 1:1 by the nearest neighbor method with a caliper of 0.2.

b SMD, standardized mean differences. SMD<0.10 presented that the variable was well matched and there was no difference between the two groups.

c BMI, body mass index; CEA, carcinoembryonic antigen; CYFRA211, cytokeratin-19 fragment 21-1; NSAIDs, nonsteroidal anti-inflammatory drugs; SCC, squamous cell carcinoma antigen; TNM, tumor-node-metastasis; VATS, video-assisted thoracic surgery.

Data are presented as the mean ± SD or *n* (%).

**FIGURE 3:**
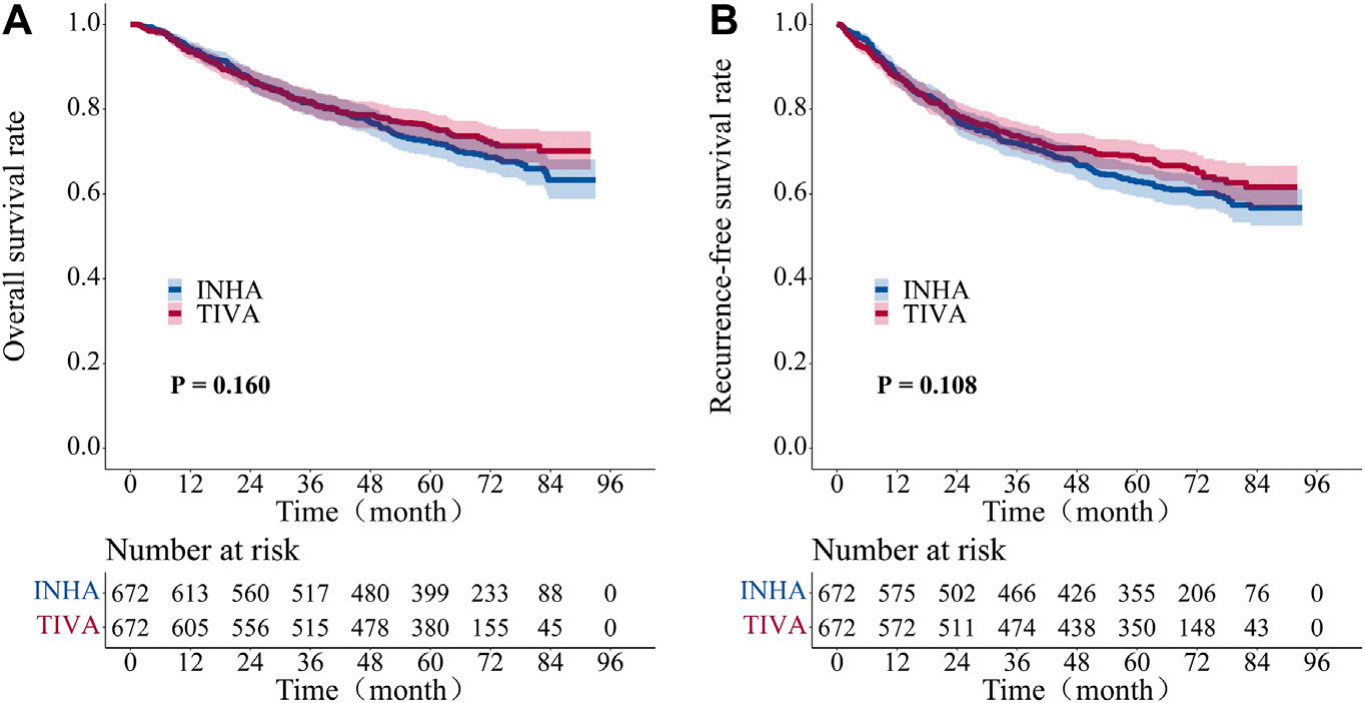
Kaplan–Meier survival curve for overall survival and recurrent-free survival in propensity-matched cohort patient. **(A)** overall survival rate. **(B)** recurrent-free survival rate. The Log-rank test was employed to evaluate the difference between the groups.

The five-year overall survival rate was 75.7% (95% CI, 72.4–79.1) in the TIVA group and 71.8% (95% CI, 68.4–75.4) in the INHA group and the five-year recurrence-free survival rates were 68.5% (95% CI, 65.0–72.2) and 62.7% (95% CI, 59.1–66.5), respectively. There were no significant differences in the overall survival (*p* = 0.160) or recurrence-free survival (*p* = 0.108) between the two groups with log-rank test (Figures 3A,B).

The Cox proportional hazards models for the overall survival and recurrence-free survival (Table 2) were constructed to compare the association between anesthesia type and cancer prognosis in the propensity-matched cohort. Multivariable Cox regression analyses showed no significant difference in the overall survival (HR, 0.86; 95% CI, 0.70–1.06; *p* = 0.158) and recurrence-free survival (HR, 0.90; 95% CI, 0.75–1.08; *p* = 0.253) between the TIVA and INHA groups. In addition, multivariable Cox regression analysis with inverse probability of treatment weighting also showed no significant association between the anesthesia type and overall survival (HR, 0.93; 95% CI, 0.76–1.12; *p* = 0.435) or recurrence-free survival (HR, 0.92; 95% CI: 0.78–1.09; *p* = 0.332).

**TABLE 2:** Multivariable Cox regression analysis for overall survival and recurrence-free survival after propensity score matching.

	Overall survival		Recurrence-free survival	
	Hazard ratio [95% CI^a^]	*p* value	Hazard ratio [95% CI]	*p* value
Anesthesia method				
INHA	reference		reference	
TIVA	0.86 (0.70, 1.06)	0.158	0.90 (0.75, 1.08)	0.253
Age (years)				
≤49	Reference		reference	
50–59	1.14 (0.80, 1.62)	0.467	1.02 (0.76, 1.37)	0.907
60–69	1.60 (1.13, 2.25)	0.007	1.23 (0.92, 1.65)	0.164
≥70	1.89 (1.17, 3.06)	0.009	1.55 (1.00, 2.40)	0.052
Sex (male)	1.26 (0.97, 1.63)	0.077	1.11 (0.88, 1.39)	0.385
BMI^a^ (kg/m^2^)				
<18.5	1.00 (0.62, 1.62)	0.985	1.06 (0.68, 1.65)	0.790
18.5–24.9	Reference		Reference	
25.0–29.9	0.71 (0.56, 0.92)	0.008	0.87 (0.70, 1.07)	0.192
≥30.0	1.29 (0.60, 2.75)	0.515	1.06 (0.52, 2.15)	0.880
Smoking	0.92 (0.73, 1.16)	0.485	0.93 (0.76, 1.15)	0.513
Comorbidities at admission				
Cardiovascular disease	0.68 (0.47, 0.97)	0.032	0.70 (0.51, 0.95)	0.022
Perioperative transfusion	1.43 (1.07, 1.91)	0.014	1.37 (1.05, 1.78)	0.020
Type of operation				
Segmental/wedge resection	3.13 (1.76, 5.56)	<0.001	2.30 (1.30, 4.07)	0.004
Lobectomy	Reference		Reference	
Pneumonectomy	1.38 (1.02, 1.89)	0.040	1.61 (1.22, 2.13)	0.001
VATS^a^	0.68 (0.52, 0.89)	0.005	0.76 (0.60, 0.96)	0.023
Complication after surgery	1.93 (1.04, 3.59)	0.038	1.90 (1.09, 3.32)	0.024
Tumor histologic type				
Adenocarcinoma	Reference		Reference	
Squamous cell carcinoma	0.79 (0.59, 1.06)	0.116	0.65 (0.50, 0.84)	0.001
Other	1.50 (1.00, 2.26)	0.050	1.31 (0.89, 1.91)	0.173
TNM^a^ stage				
Ⅰ	Reference		Reference	
Ⅱ	2.62 (1.94, 3.53)	<0.001	2.23 (1.72, 2.90)	<0.001
Ⅲ	4.16 (3.17, 5.47)	<0.001	2.92 (2.30, 3.72)	<0.001
SCC^a^ (1–1.5 ng/ml)	0.95 (0.70, 1.29)	0.741	1.03 (0.78, 1.37)	0.820
CEA^a^ (0–5 ng/ml)	1.49 (1.18, 1.87)	0.001	1.61 (1.32, 1.97)	<0.001
CYFRA211^a^ (0–3.3 ng/ml)	1.25 (0.99, 1.57)	0.060	1.25 (1.02, 1.54)	0.030
Adjuvant radiotherapy	2.27 (1.70, 3.04)	<0.001	3.82 (2.99, 4.88)	<0.001
Adjuvant chemotherapy	0.74 (0.58, 0.95)	0.016	1.21 (0.98, 1.50)	0.078

a BMI, body mass index; CEA, carcinoembryonic antigen; CI, confidence interval; CYFRA211, cytokeratin-19 fragment 21-1; SCC, squamous cell carcinoma antigen; TNM, tumor-node-metastasis; VATS, video-assisted thoracic surgery.

Data are presented as hazard ratio [95% CI].

**FIGURE 4:**
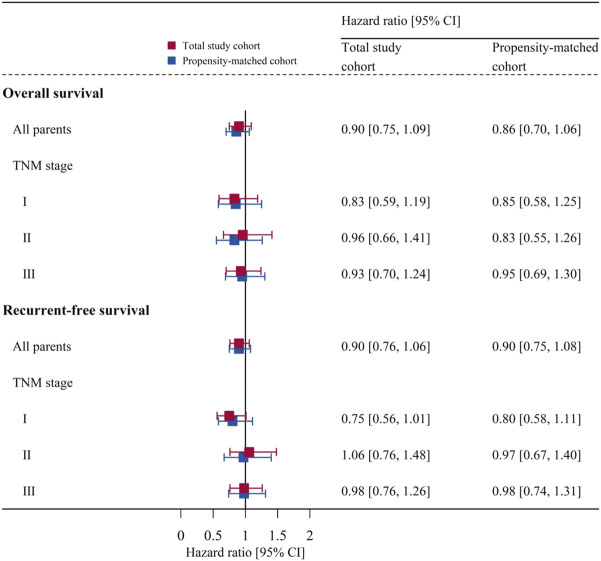
Subgroup analyses for each TNM stage of NSCLC: association of anesthesia type with overall survival or recurrent-free survival before and after matching. Data are presented as hazard ratio [95% CI]. CI, confidence interval; TNM, tumor-node-metastasis.

The subgroup analyses showed that there were no significant differences in the overall survival or recurrence-free survival between the two groups in each TNM stage of NSCLC (Figure 4).

The independent risk factors for cancer recurrence or all-cause mortality included age ≥60 years old, male patients, perioperative blood transfusion, segmental/wedge resection and pneumonectomy, thoracotomy, postoperative complications, lung adenocarcinoma, TNM stages, elevated CEA and CYFRA211 values, and postoperative radiotherapy (Table 3).

**TABLE 3:** Multivariable Cox regression analysis for overall survival and recurrence-free survival in the total study cohort.

	Overall survival		Recurrence-free survival	
	Hazard ratio [95% CI^a^]	*p* value	Hazard ratio [95% CI]	*p* value
Anesthesia method				
INHA	reference		reference	
TIVA	0.90 (0.75, 1.09)	0.292	0.90 (0.76, 1.06)	0.216
Age (years)				
≤49	Reference		Reference	
50–59	1.14 (0.86, 1.53)	0.368	0.92 (0.73, 1.17)	0.504
60–69	1.43 (1.07, 1.91)	0.015	0.99 (0.78, 1.25)	0.921
≥70	1.66 (1.13, 2.44)	0.010	1.30 (0.93, 1.83)	0.121
Sex (male)	1.47 (1.18, 1.82)	<0.001	1.29 (1.07, 1.55)	0.008
BMI^a^ (kg/m^2^)				
<18.5	1.04 (0.68, 1.60)	0.855	1.09 (0.74, 1.61)	0.662
18.5–24.9	Reference		Reference	
25.0–29.9	0.77 (0.62, 0.95)	0.014	0.87 (0.73, 1.04)	0.115
≥30.0	0.86 (0.46, 1.62)	0.644	0.92 (0.54, 1.57)	0.756
Smoking	0.95 (0.78, 1.15)	0.588	0.94 (0.79, 1.12)	0.475
Comorbidities at admission				
Cardiovascular disease	0.77 (0.56, 1.06)	0.113	0.76 (0.57, 1.01)	0.056
Perioperative transfusion	1.45 (1.12, 1.87)	0.004	1.33 (1.06, 1.68)	0.015
Type of operation				
Segmental/wedge resection	2.83 (1.66, 4.82)	<0.001	2.24 (1.34, 3.74)	0.002
Lobectomy	Reference		Reference	
Pneumonectomy	1.33 (1.02, 1.74)	0.036	1.52 (1.20, 1.92)	<0.001
VATS^a^	0.76 (0.60, 0.96)	0.022	0.82 (0.67, 1.00)	0.049
Complication after surgery	1.71 (1.02, 2.85)	0.041	1.84 (1.19, 2.85)	0.006
Tumor histologic type				
Adenocarcinoma	reference		Reference	
Squamous cell carcinoma	0.80 (0.63, 1.02)	0.075	0.64 (0.52, 0.80)	<0.001
Other	1.56 (1.09, 2.23)	0.016	1.32 (0.94, 1.84)	0.111
TNM^a^ stage				
Ⅰ	Reference		Reference	
Ⅱ	2.22 (1.72, 2.86)	<0.001	1.86 (1.49, 2.31)	<0.001
Ⅲ	3.87 (3.06, 4.89)	<0.001	2.59 (2.12, 3.18)	<0.001
SCC^a^ (1–1.5 ng/ml)	0.89 (0.68, 1.17)	0.408	1.05 (0.83, 1.34)	0.657
CEA^a^ (0–5 ng/ml)^a^	1.42 (1.17, 1.74)	<0.001	1.58 (1.33, 1.87)	<0.001
CYFRA211^a^ (0–3.3 ng/ml)	1.25 (1.03, 1.52)	0.026	1.23 (1.03, 1.46)	0.019
Adjuvant radiotherapy	2.08 (1.62, 2.67)	<0.001	3.70 (3.02, 4.54)	<0.001
Adjuvant chemotherapy	0.75 (0.61, 0.92)	0.007	1.26 (1.05, 1.51)	0.013

a BMI, body mass index; CEA, carcinoembryonic antigen; CI, confidence interval; CYFRA211, cytokeratin-19 fragment 21-1; SCC, squamous cell carcinoma antigen; TNM, tumor-node-metastasis; VATS, Video-assisted thoracic surgery.

Data are presented as the hazard ratio [95% CI].

## Discussion

This retrospective study indicated that the propofol-based TIVA was not beneficial to the prognosis of cancer patients than the sevoflurane-based INHA in patients with NSCLC. The independent risk factors included age ≥60 years old, male patients, TNM stage, elevated CEA and CYFRA211 values, perioperative blood transfusion, thoracotomy, wedge resection, postoperative complications, and postoperative radiotherapy.

Perioperative factors, such as surgery-induced neuroendocrine stress, injury-related inflammation, hyperglycaemia, and hypothermia responsible for immunosuppression, which can foster a potentially protumor environment that may facilitate distal seeding of circulating tumor cells, the growth of micrometastases into established clinical metastases, or both (Jeon et al., 2020). This kind of protumor environment may be further aggravated by the administration of anesthetics that themselves have a potential direct effect on the cancer cell biology and indirectly modulating effects. Indeed, propofol promoted NK cell cytotoxicity and potentiated the expression of CD28 on peripheral T-helper cells (Xu et al., 2020). In contrast, volatile anesthetics inhibited NK cell-mediated cytotoxicity, induced T-lymphocyte apoptosis, and upregulated tumorigenic modulator tumorigenic growth factors, VEGF, and HIF-1 (Buckley et al., 2014; Shi et al., 2015; Iwasaki et al., 2016; Lim et al., 2018). Sevoflurane caused the accumulation of intracellular reactive oxygen species by activating the PI3K and MAPK signaling pathways, and upregulating the expression of MMPs (Fan et al., 2019). Li et al. (2020) found that sevoflurane anesthesia led to significantly more lung metastasis than propofol in mice models with surgery of primary tumors. Similarly, a retrospective analysis by Hayasaka et al. (2021) revealed that in 230 patients, who had surgery with pathological stage I NSCLC, the five-year recurrence-free survival rates in the TIVA group was 91.7%, which was significantly higher than that in the INHA group (77.4%), indicating that propofol anesthesia may improve the prognosis of early NSCLC patients. However, in line with previous retrospective studies (Oh et al., 2018; de La Motte Watson et al., 2021), our retrospective analysis revealed that propofol-based TIVA was not associated with significantly better survival when compared to INHA in patients with surgically resected p-stage I-III NSCLC. In our study, the five-year recurrence-free survival rate was 68.5% in the TIVA group and 62.7% in the INHA group. Previous studies have found that the five-year cancer-specific survival of patients with lung cancer in the TIVA group was 68.1%, and that in the inhalation group was 70.8%. But patients who underwent thoracoscopic surgery were not included in this study (de La Motte Watson et al., 2021). There are several possible explanations for these findings. First, in our patients, propofol was also used in the inhalation group for anesthesia induction. The stress response and immunosuppression caused by the operation stimulated the progression of tumor cells during the perioperative period. Propofol was reported to have less immunosuppression and has a lower inflammatory response than volatile drugs during the perioperative period (Lee et al., 2022). This may reduce opioid use for pain relief and may have favorable to contribute outcomes (Moorthy et al., 2021). Therefore, the net effects of propofol and inhalational agent sevoflurane on the oncologic outcomes in our patients remain unknown and this was the case for many studies, even clinical trials (Ishikawa et al., 2020). Second, sevoflurane was found to suppress lung cancer cells while it promoted renal cancer cell growth in *in vitro* model (Ciechanowicz et al., 2018). If those findings were corroborated in clinical settings, then its differential effects may contribute no different outcomes between two anesthetics based anesthesia. Third, due to the retrospective nature of our study, we cannot comment on study power but it is very likely underpower *per se*. Finally, there are many factors affecting the oncological outcomes following surgery and anesthetic use is likely one of them and thus, negative findings are not surprised.

VATS lobectomy have been performed increasingly, as an alternative to open thoracotomy in the early-stage NSCLC, and the advantages include smaller incisions, less postoperative pain and complications, and shorter length of hospitalization (Boffa et al., 2014; Oda et al., 2019). However, VATS removed fewer lymph nodes, especially N2 nodes, compared with thoracotomy, thus this concern would heighten the risk of leaving residual tumors at the surgical margin, and an insufficient lymph node dissection (Boffa et al., 2012; Decaluwé et al., 2016). In the study, VATS was associated with improved survival, due to better postoperative quality of life and overall survival rates compared to open thoracotomy. Despite there being no demonstrable significant difference in the postoperative complications in this study, this is most likely attributable to a combination of factors including a significantly shorter hospital stay, decreased postoperative pain, improved pulmonary toilet, and earlier chest tube removal (Yang et al., 2017). The study did not exclude patients who received the wedge resection of tumor. The pulmonary wedge resection causes a compression on the pulmonary tissue and the tumor, which may result in the shedding of tumor cells into the blood (Li et al., 2015). In addition, perioperative allogeneic blood transfusions have a dose-dependent relationship with a shorter disease-free time interval and early recurrence in patients with stage I–II NSCLC, and a peak effect occurred at six units (Latif et al., 2019). pRBC transfusions immunoedited in patients with NSCLC due to allogenic cells and antigens that divert and disrupt the balance between immune surveillance and cancer progression (Muszynski et al., 2017). Thus, the type of surgery and blood transfusions were independent factors of shorter long-term survival in patients undergoing NSCLC resection.

In the study, various variables were also found to be significantly associated with mortality including older age, TNM stage, and adjuvant radiotherapy. Our subgroup analyses showed that there were no significant differences in the overall survival or recurrence-free survival after the propensity-matched cohort, in each TNM stage of NSCLC. Studies showed that the risk of poor survival increases significantly with increasing age (Vogelaar et al., 2015), ASA physical status, and poor preoperative functional status (Wigmore et al., 2016), and neoadjuvant chemotherapy has prolonged the prognosis of tumor (Aloia et al., 2014).

There are limitations to our study. First, this is a single-center retrospective observational study, patients were not randomly allocated, and selection bias may exist, although Cox regression analysis was performed to reduce the potential confounding effect of each variable. The sample size was further reduced while balancing the baseline characteristics between the two groups *via* propensity score matching. Second, the longest follow-up period of our study was eight years, and there could have been advances in surgical, anesthetic, and neoadjuvant chemotherapy management. These changes were not adequately considered. Finally, patients in the both groups received propofol during anesthesia induction in our study, which may mask the effect of sevoflurane. All these factors need to be addressed in the future prospective multicenter study.

## Conclusion

Our study showed no benefit for propofol-based TIVA, in comparison with sevoflurane-based INHA, in terms of five-year overall survival and recurrence-free survival after NSCLC surgery. Large-sample size, well-designed prospective multicenter studies are necessary to clarify this association of anesthetics and oncologic outcomes.

## Data Availability

The raw data supporting the conclusions of this article will be made available by the authors, without undue reservation.
